# Chromosome-Scale Genome for a Red-Fruited, Perpetual Flowering and Runnerless Woodland Strawberry (*Fragaria vesca*)

**DOI:** 10.3389/fgene.2021.671371

**Published:** 2021-07-16

**Authors:** Elizabeth I. Alger, Adrian E. Platts, Sontosh K. Deb, Xi Luo, Shujun Ou, Yao Cao, Kim E. Hummer, Zhiyong Xiong, Steven J. Knapp, Zhongchi Liu, Michael R. McKain, Patrick P. Edger

**Affiliations:** ^1^Department of Horticulture, Michigan State University, East Lansing, MI, United States; ^2^Department of Biological Sciences, University of Alabama, Tuscaloosa, AL, United States; ^3^Department of Forestry and Environmental Science, Shahjalal University of Science and Technology, Sylhet, Bangladesh; ^4^Department of Cell Biology and Molecular Genetics, University of Maryland, College Park, MD, United States; ^5^Department of Ecology, Evolution, and Organismal Biology, Iowa State University, Ames, IA, United States; ^6^College of Life Science, Inner Mongolia University, Hohhot, China; ^7^USDA ARS National Clonal Germplasm Repository, Corvallis, OR, United States; ^8^Department of Plant Sciences, University of California, Davis, Davis, CA, United States; ^9^Genetics and Genome Sciences Program, Michigan State University, East Lansing, MI, United States

**Keywords:** *Fragaria vesca*, woodland strawberry, runnering, fruit color, perpetual flowering

Strawberry (*Fragaria sp*.) is emerging as an important model system for the family Rosaceae, which includes several fruit crop species (e.g., cherries, peaches, and strawberries). *Fragaria* research has largely utilized the woodland strawberry species, *Fragaria vesca*, due to it's small size, short generation time, and ease of transformation. In addition to its ease of use, *F. vesca* is also the closest extant relative of the diploid progenitor for the dominant subgenome in the allo-octoploid cultivated garden strawberry (*F*. × *ananassa*) making it an useful organism for studying polyploidy in cultivated strawberry (Shulaev et al., 2011; Alger et al., 2018; Alonge et al., 2019; Edger et al., 2019). The *F. vesca* accession “Hawaii-4” has been widely used in previous genetic studies. The genome was first sequenced in 2011 using short read sequencing (Shulaev et al., 2011) and then improved in 2014 using linkage maps (Tennessen et al., 2014). A chromosome-scale version of the “Hawaii-4” genome was assembled using a combination of long read and short read sequencing data in 2018 (Edger et al., 2018) and a revised annotation released in 2019 (Li et al., 2019). However, this single accession fails to capture the agriculturally valuable genetic and phenotypic diversity of *F. vesca* (Shulaev et al., 2011; Tennessen et al., 2014; Alger et al., 2018). The diverse accessions of *F. vesca* exhibit a wide range of phenotypes, including several traits relevant to breeding programs such as flowering time, fruit color, and runner production.

In strawberry, the axillary meristem can develop into either daughter-plant producing runners or into fruit-bearing shoots (Costes et al., 2014; Heide et al., 2015; Hytönen and Kurokura, 2020). Strawberry cultivars are maintained and grown from these runners rather than from seeds, as runners allow for easy clonal propagation (HytÖnen et al., 2004; Costes et al., 2014; Heide et al., 2015; Hytönen and Kurokura, 2020). However, runnering is also associated with a decrease in fruit yield as increased runner production reduces the number of fruit-bearing shoots (Gaston et al., 2013; Heide et al., 2015; Tenreira et al., 2017). Therefore, while the fruit is the consumer product, runners are also essential for strawberry propagation. Beyond influencing fruit yields, runners can also grow quickly and abundantly, resulting in the need for frequent trimming for plant maintenance. With these considerations, better understanding genetic factors influencing the switch between inflorescence and runner growth would be a valuable resource for the strawberry community.

*F. vesca* accessions range from completely runnerless to extremely high runnering, allowing for further investigation into this important trait in strawberry (Tenreira et al., 2017; Caruana et al., 2018). Here we present a high quality genome of an *F. vesca* accession, CFRA 2339 (PI 698244), which phenotypically differs from Hawaii-4 in two important traits: fruit color and runner production (Figures 1A,B). Hawaii-4 has yellow fruit and produces runners while CFRA 2339 produces red fruit and is runnerless (Hawkins et al., 2016; Tenreira et al., 2017; Caruana et al., 2018; Castillejo et al., 2020). Both accessions are perpetual flowering instead of the seasonal flowering displayed by other accessions (Iwata et al., 2012). This genome will serve as a valuable new resource for the strawberry and the larger Rosaceae community, allowing for the identification of the genetics controlling runner production as well as fruit color in a diploid model organism (Caruana et al., 2018; Castillejo et al., 2020).

**Figure 1:**
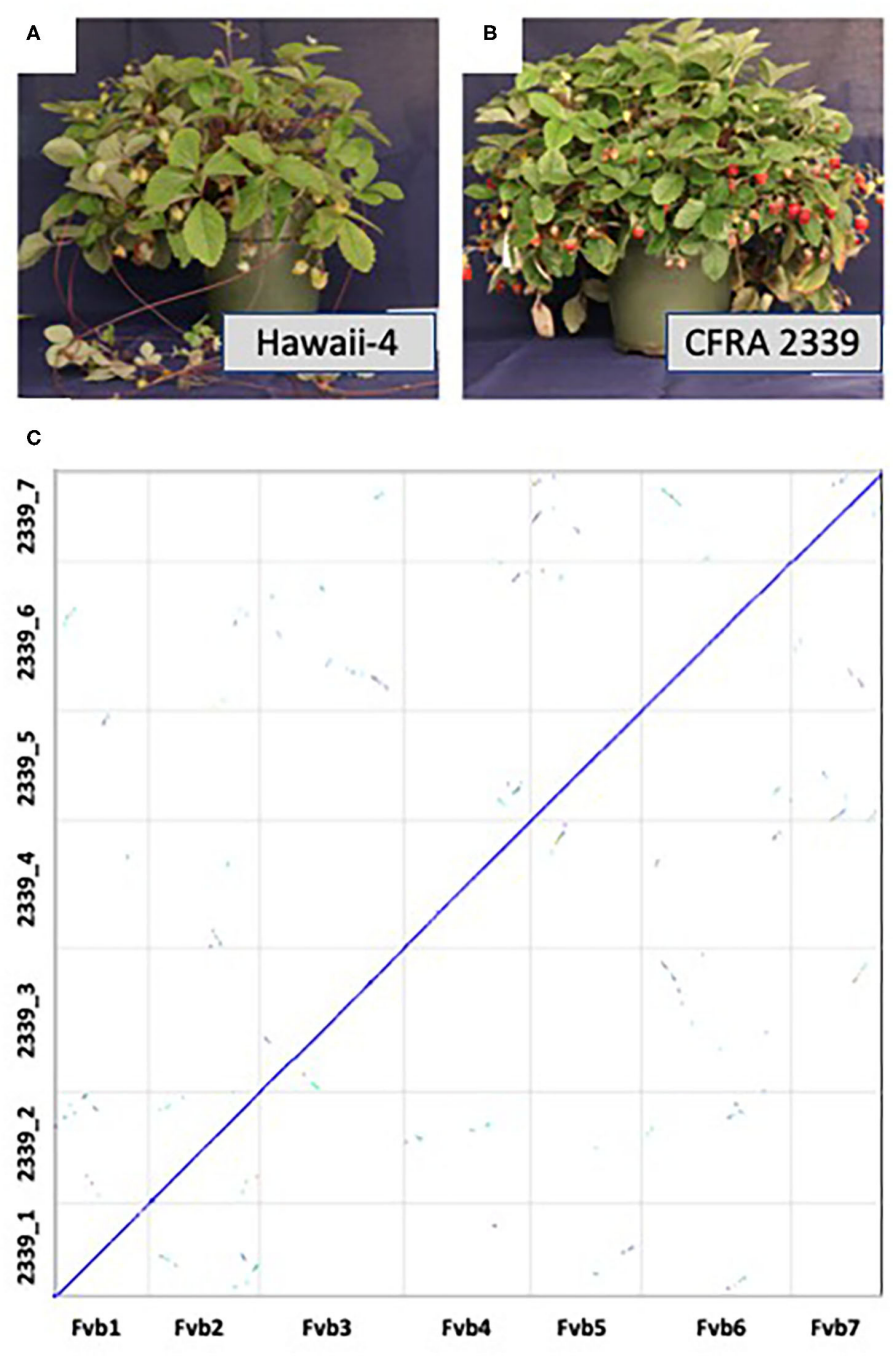
Pictures of mature *Fragaria vesca* Hawaii-4 **(A)** and CFRA 2339 **(B)**. Hawaii-4 produces runners and yellow fruit, while CFRA 2339 is runnerless and produces red fruit. **(C)** depicts synteny between the final assembly of *F. vesca* Hawaii-4 (x-axis) and CFRA 2339 (y-axis). Synteny analysis was performed in CoGe [Comparative Genomics, https://genomevolution.org/coge/ (accessed April 10, 2021)] with coloring coding depicting the number of synonymous substitutions. Syntenic orthologous regions between the two accessions are shown on the diagonal in blue, and duplicated genes retained from a whole-genome triplication event [At-gamma (Bowers et al., 2003)] are off-diagonal in other colors. This analysis and figure can be regenerated here: https://genomevolution.org/r/1hdms.

The aim of this paper is to expand the resources of the model species *F. vesca* by providing a second high quality genome from an accession with different phenotypic traits compared to the current high quality *F. vesca* Hawaii-4 genome (Edger et al., 2018). To accomplish this, we combined long-read Oxford Nanopore sequencing and high coverage short read Illumina sequencing. We generated ~2.3 million Nanopore reads collectively, totalling over 30 Gb, providing >120x coverage for the CFRA 2339 genome, and exhibiting an N50 length of 34.1kb and a maximum length of 311kb. The raw Nanopore reads were then corrected and assembled using the Canu assembler (Koren et al., 2017). *Fragaria vesca* has seven chromosomes. Chromosomes 1, 5, and 7 were captured by a single contig, 2, 4, and 6 were split between two contigs, and chromosome 3 was split among three contigs, thus requiring minimal further scaffolding (Supplementary Figure 1; Supplementary Table 1). RagTag was then used to correct misassemblies and merge scaffolds into pseudomolecules using Hawaii-4 as a reference and then polished with two rounds of Pilon with over 35.5 Gb Illumina data (Walker et al., 2014). The final assembly spanned 229.5Mb across 311 contigs with an N50 length of 29.6 Mb (Figure 1C; Supplementary Table 2). Seven pseudomolecules were obtained for the CFRA 2339 genome (Figure 1C).

The heterozygosity of CFRA 2339 was estimated using Illumina genomic data and Jellyfish (Marçais and Kingsford, 2011) with K=31, and the histogram file processed with genomescope (Vurture et al., 2017). The results suggest that CFRA 2339 has a heterozygosity level of roughly 0.096% (Supplementary Figure 2). There is an additional ~10.2Mb of unanchored sequences not present in the seven pseudomolecules of CFRA 2339, which is equivalent to ~4.6% of anchored sequences. Given the syntenic coverage across the Hawaii-4 (Figure 1C), some subset of these unanchored sequences are likely haplotype variants. This needs to be further investigated in future studies, including with other current and future long read sequencing and assembly approaches. Furthermore, there is similar coverage across the putative centromere and ribosomal DNA regions, and may also extend to the telomere end of chromosome as previously reported for the Hawaii-4 genome (Edger et al., 2018; Li et al., 2018).

The genome was annotated using the MAKER annotation pipeline using gene evidence from a broad SRA *F. vesca* dataset, RNAseq datasets generated from CFRA 2339 tissues, as well as gene and protein evidence from the *F. vesca* Hawaii-4 v4 annotation and the UniprotKB database (Holt and Yandell, 2011; UniProt Consortium, 2019). We identified 30,349 gene models with 64% having a known Pfam domain (Supplementary Table 3). The overall number of annotated genes is similar to the Hawaii-4 genome annotation (Supplementary Table 3). The most recent version of the Hawaii-4 genome annotated 34,009 genes (Li et al., 2019). The Benchmarking Universal Single-Copy Orthologs (BUSCO) with the eudicot database (eudicot_odb10) was used to estimate the completeness of the genome assembly and the CFRA 2339 genome annotation quality (Simão et al., 2015) (Supplementary Table 3). The genome was found to have 96% of the core genes in the BUSCO eudicots dataset, supporting a high quality genome assembly and annotation. Transposable elements (TEs) were annotated using the Extensive *de novo* TE Annotator (EDTA) (Ou et al., 2019) (Supplementary Table 4). EDTA found that TEs comprise ~29.7% of the *F. vesca* CFRA 2339 genome, with long-terminal-repeat retrotransposons (LTR-RT) being the most abundant and accounting for ~16% of the overall genome. The amount of annotated TEs in the CFRA 2339 genome is similar to the ~29.3% of TE content previously annotated in the Hawaii-4 genome (Edger et al., 2018) (Supplementary Table 5).

Based on comparative genomic analyses (Figure 1C), there is consistent coverage of the CFRA 2339 genome across the Hawaii-4 genome. We were able to identify syntelogs for roughly 94.7% of genes or all but 1,501 genes encoded on the seven pseudomolecules of Hawaii-4. We investigated these 1,501 missing genes and found that the vast majority (1166 total) had syntenic flanking genes. In other words, these missing genes are single loci with flanking genes present in both genomes. We also found 134 instances of two adjacent genes missing (268 genes total) and 13 instances of three adjacent genes missing (39 genes total). This suggests that these are likely either genes exhibiting presence-absence variation and/or were possibly not assembled or annotated. The remaining missing genes were one instance of four adjacent missing genes, two instances of five adjacent missing genes, one instance of six adjacent missing genes and one instance of eight adjacent missing genes. None of these missing gene blocks occurred at the boundaries of contigs. The amount of PAV identified between these two accessions is within the range previously reported for other species (e.g., Golicz et al., 2016; Gordon et al., 2017; Yocca et al., 2021). The verification of these PAV loci is something that needs to be explored in greater detail as part of future strawberry pangenome studies. It's worth noting that missing gene models may be due to remnant sequencing errors from nanopore that were unable to be removed with Illumina polishing.

The woodland *Fragaria vesca* exhibits natural variation for several important agronomic traits including fruit color, flowering, and runnering. Previous molecular genetic studies have shown that variation in each of these traits can be controlled by single loci. For example, a G-to-C SNP in the *FveMYB10* coding region was responsible for the yellow fruit color in several *F. vesca* accessions (Hawkins et al., 2016) (Table 1; Supplementary Figure 3). Also, as an important hormone promoting runner formation, GA is synthesized by several enzymes including gibberellin 20-oxidase encoded by *FveGA20ox* genes; a 9-bp-deletion in the *FveGA20ox4* gene was found to be responsible for the runnerless phenotype of *F. vesca* (Tenreira et al., 2017) (Table 1; Figure 1). Caruana et al. found a nonsense mutation in the DELLA protein coded by *FveRGA1*, which was responsible for the constitutive runnering even when the *FveGA20ox4* gene is mutated (Caruana et al., 2018). Finally, some *F. vesca* varieties are perpetual flowering, this phenotype was shown to be caused by a 2-bp deletion in the first exon of *FveTFL1*, coding for a repressor of flowering (Iwata et al., 2012; Koskela et al., 2012) (Table 1).

**Table 1:** Genotype and phenotype comparisons among several *F. vesca* accessions with different fruit, runner, and flowering traits.

**Trait**	**SD *F. vesca* (PI551792)**	**Hawaii-4**	**Rugen**	**Yellow wonder (5AF7)**	**CFRA 2339**
Species	*Fragaria vesca subsp. vesca*	*Fragaria vesca subsp vesca f. alba*	*Fragaria vesca subsp vesca f. semperflorens*	*Fragaria vesca subsp vesca f. alba*	*Fragaria vesca subsp. vesca f. semperflorens*
Runnering	Prolific runner	Prolific runners	Runnerless	Runnerless	Runnerless
*GA20ox4*	NA	WT	−9 bp	−9 bp	−9 bp
Fruit color	Red	Yellow	Red	Yellow	Red
*MYB10*	NA	G134C	WT	G134C	WT
Flowering	Seasonal flowering	Perpetual flowering	Perpetual flowering	Perpetual flowering	Perpetual flowering
*TFL1*	WT	−2 bp	−2 bp	−2 bp	−2 bp

*We analyzed the sequence of MYB10, GA20ox4, TFL1, and RGA1 in the new genome of CFRA 2339 by aligning their CDS with Hawaii-4 reference genome (Edger et al., 2018). We found that CFRA 2339 is wild type (WT) for MYB10 for red berries, but carries the 9-bp deletion in GA20ox4 and the wild type gene for RGA1 responsible for the runnerless phenotype, as well as the 2-bp mutation in the TFL1 gene responsible for perpetual flowering. This is consistent with CFRA 2339 red fruit color, runnerless, and perpetual flowering phenotypes. Data from the other F. vesca accessions were obtained from these studies (Iwata et al., 2012; Koskela et al., 2012; Hawkins et al., 2016; Tenreira et al., 2017; Caruana et al., 2018)*.

The CFRA 2339 accession produces red fruit, flowers perpetually, and is runnerless. We analyzed the sequences of *MYB10* (Hawkins et al., 2016), *GA20ox4* (Tenreira et al., 2017), *TFL1* (Iwata et al., 2012; Koskela et al., 2012) and *RGA1* (Caruana et al., 2018) in the newly assembled genome of CFRA 2339 by aligning their CDS with the orthologs from the Hawaii-4 reference genome (Edger et al., 2018). We found that CRFR 2339 has the *MYB10* variant for red berries, but carries the 2-bp mutation in the *TFL1* gene responsible for perpetual flowering (Table 1). Further, the 9-bp deletion in *GA20ox4* (but the functional full-length variant for *RGA1)* explains the runnerless phenotype (Table 1). The genotypes at these loci are consistent with CFRA 2339 red fruit color, runnerless, and perpetual flowering phenotypes.

The genome described here for *F. vesca* CFRA 2339 will be a valuable new resource for the strawberry community. Furthermore, being runnerless, CFRA 2339 does not require the frequent trimming that is necessary for most other accessions, but can still be propagated easily by splitting and replanting the crown. The accession does share the perpetual flowering trait with Hawaii-4, leading to high flower and fruit production.

## Methods

The *Fragaria vesca* accession CFRA 2339 (PI 698244) was acquired from the USDA ARS National Clonal Germplasm Repository in Corvallis, Oregon. Plants were grown in a growth chamber under standard conditions. High molecular weight DNA from CFRA 2339 for Oxford Nanopore sequencing was extracted from young leaves after 72 h of dark treatment using the method described in Vaillancourt and Buell (Vaillancourt and Robin Buell, 2020). RNA was extracted from young leaves, old leaves, dark-treated young leaves, dark-treated old leaves, methyl jasmonate-treated leaves, roots, shoots, flowers, and flowers before anthesis. All tissues were collected from CFRA 2339 plants at 12pm and extracted using MagMAX^TM^-96 Total RNA Isolation Kit (Thermo Fisher).

Two libraries were prepared from the input gDNA using the Oxford Nanopore LSK109 kit. Each of the two libraries were loaded on one Oxford Nanopore FLO-MIN106D (R9.4.1) flow cell and sequenced on a GridION x5 running GridION release 19.12.2. Realtime base calling was performed by guppy basecaller v3.2.8, High Accuracy mode. After ~24 h of sequencing the run was paused to flush and reload the flow cell with additional library; this pause, flush and reload was repeated at 48 h. The total run time was 72 h.

Raw Oxford Nanopore reads were trimmed of adapters using Porechop v.0.2.3 with an adapter threshold of 85% identity, an extra end trim of 10 base pairs after identification of adapters, a middle threshold of 80% identity, and all other parameters at default. All reads with a final length of less than 10,000 base pairs after trimming were removed. Canu v1.9 (Koren et al., 2017, 2018) was then used to correct, trim, and assemble adapter-trimmed reads assuming a genome size of 250 Mb and under default parameters. The assembly of the CFRA 2339 genome was compared to that of a well assembled diploid reference (*F. vesca* “Hawaii-4”), using COGE SynMap supplemented with an Hawaii-4 (Edger et al., 2018) gff3 annotation and a conda installation of RagTag (Haug-Baltzell et al., 2017; Edger et al., 2018; Alonge et al., 2019). Two major inversions were noted in the syntenic map between the two assemblies and these regions were further explored for spanning reads that supported either the CFRA 2339 or Hawaii-4 structure. Ultimately all regions where structures differed were found to be at assembly weak-spots comprised of similar repetitive regions in the CFRA 2339 assembly with only ambiguous supporting alignments. Consequently, we used RagTag in misassembly correction mode to harmonize the inversions followed by further consolidation using RagTag in scaffolding mode to merge the remaining fragmented scaffolds into pseudomolecules, generating a genome guided assembly based on the Hawaii-4 v4 reference assembly (Alonge et al., 2019). The hybrid corrected assembly was then polished with two rounds of Illumina paired-end data aligned with BWA (version 0.7.13, mode: mem) using Pilon (version 1.23) with default parameters (Li, 2013; Walker et al., 2014).

The polished hybrid assembly was annotated with Maker (2.31.10) in eukaryotic mode using several EST datasets: ESTs generated from a Trinity (v2.11.0) transcriptome assembly guided by the polished reference and informed by RNAseq data from a broad SRA Vesca dataset; ESTs generated from a Trinity assembly of RNAs generated from CFRA 2339 young leaf, old leaf, young dark-treated leaf, old dark treated leaf, methyl jasmonate-treated leaves, flowers, flower before anthesis, stems, and root tissues; ESTs were also introduced from previous *F. vesca* transcript annotations including FV4.0.a2 and 4.0.a1 (Grabherr et al., 2011; Edger et al., 2018; Li et al., 2018). Annotation also used a protein homology dataset from the *F. vesca* Hawaii-4 v4 and a similarly derived repeat masking library. Several gene prediction Markov models were also introduced including a SNAP model trained essentially as described by the authors, the Genemark eukaryotic HMM (version 3) and Augustus (version 3.3.3) hmm models generated using Augustus training on 1,000 gene sequences derived from the v4.0.a1 gff3 annotation together with 1kb flanking regions (Lukashin and Borodovsky, 1998; Korf, 2004; Stanke et al., 2008).

The CDS sequences of *MYB10* (Caruana et al., 2018), *GA20ox4* (HytÖnen et al., 2004), and *TFL1* (Li et al., 2018; Castillejo et al., 2020) from the new CFRA 2339 genome and the Hawaii-4 reference genome were used to identify differences in these four genes between the two accessions along with results generated in previous papers (Koskela et al., 2012; Hawkins et al., 2016).

## Data Availability Statement

The genome assembly, annotations, and other supporting data are available on the Genome Database for Rosaceae (Koren et al., 2018; GDR, Genome Database for Rosaceae, 2021) and the CyVerse CoGe (Haug-Baltzell et al., 2017) platform. The raw sequence data have been deposited in the Short Read Archive under NCBI BioProject ID PRJNA695578.

## Author Contributions

EA and PE designed the research. EA, AP, SD, XL, YC, ZX, SK, KH, ZL, MM, and PE performed research and/or analyzed data. EA wrote the paper. All authors reviewed the manuscript.

## Conflict of Interest

The authors declare that the research was conducted in the absence of any commercial or financial relationships that could be construed as a potential conflict of interest.

## Abbreviations

bp: base pair
BUSCO: Benchmarking Universal Single-Copy Orthologs
kb: kilo base
Mb: mega base
TE: transposable element
Syntelog: syntenic ortholog.
