# Supplementary figures and images for: Chromosome-Scale Genome for a Red-Fruited, Perpetual Flowering and Runnerless Woodland Strawberry (Fragaria vesca)
Source: Front Genet. 2021 Jul 16;12:671371. doi: 10.3389/fgene.2021.671371 (PMC8323839; doi:10.3389/fgene.2021.671371)

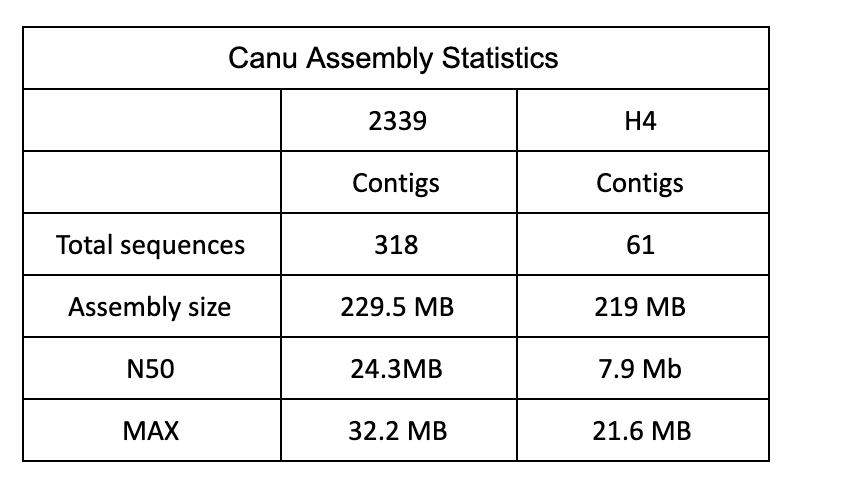


**Supplemental Table 1**: Canu Assembly statistics of CFRA 2339 and Hawaii-4.

Supplement: Supplementary file 1 [file Table_1.docx]

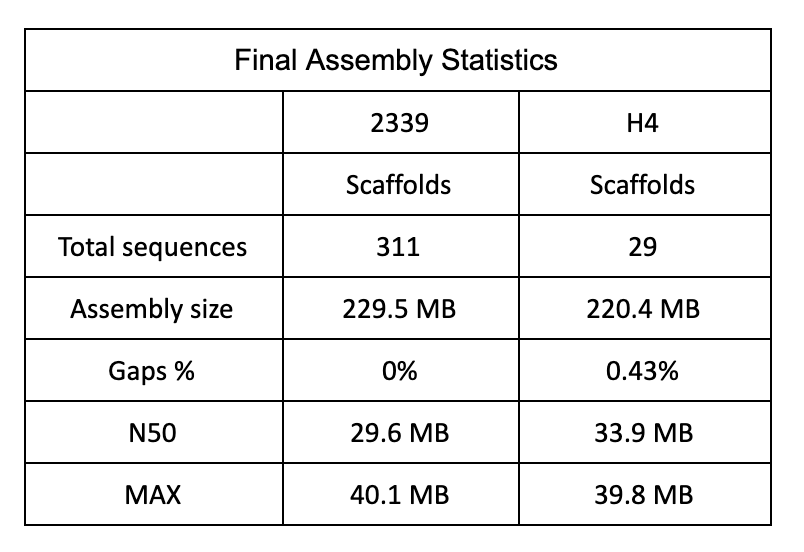


**Supplemental Table 2**: Final assembly statistics of CFRA 2339 and Hawaii-4.

Supplement: Supplementary file 2 [file Table_2.docx]

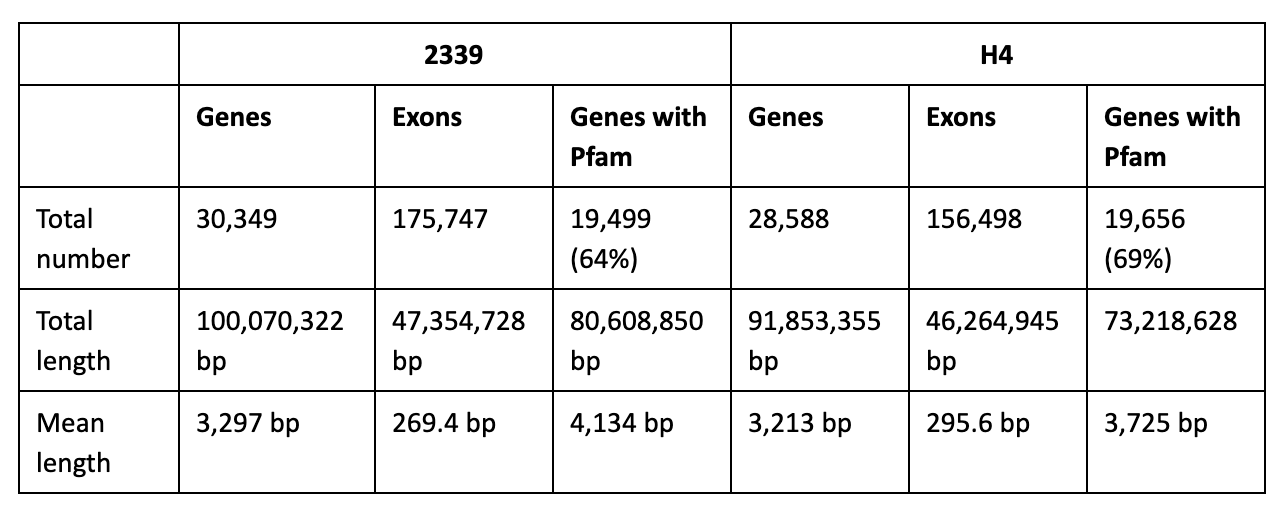


**Supplemental Table 3**: Gene features number and length statistics of CFRA 2339 and Hawaii-4.

Supplement: Supplementary file 3 [file Table_3.docx]

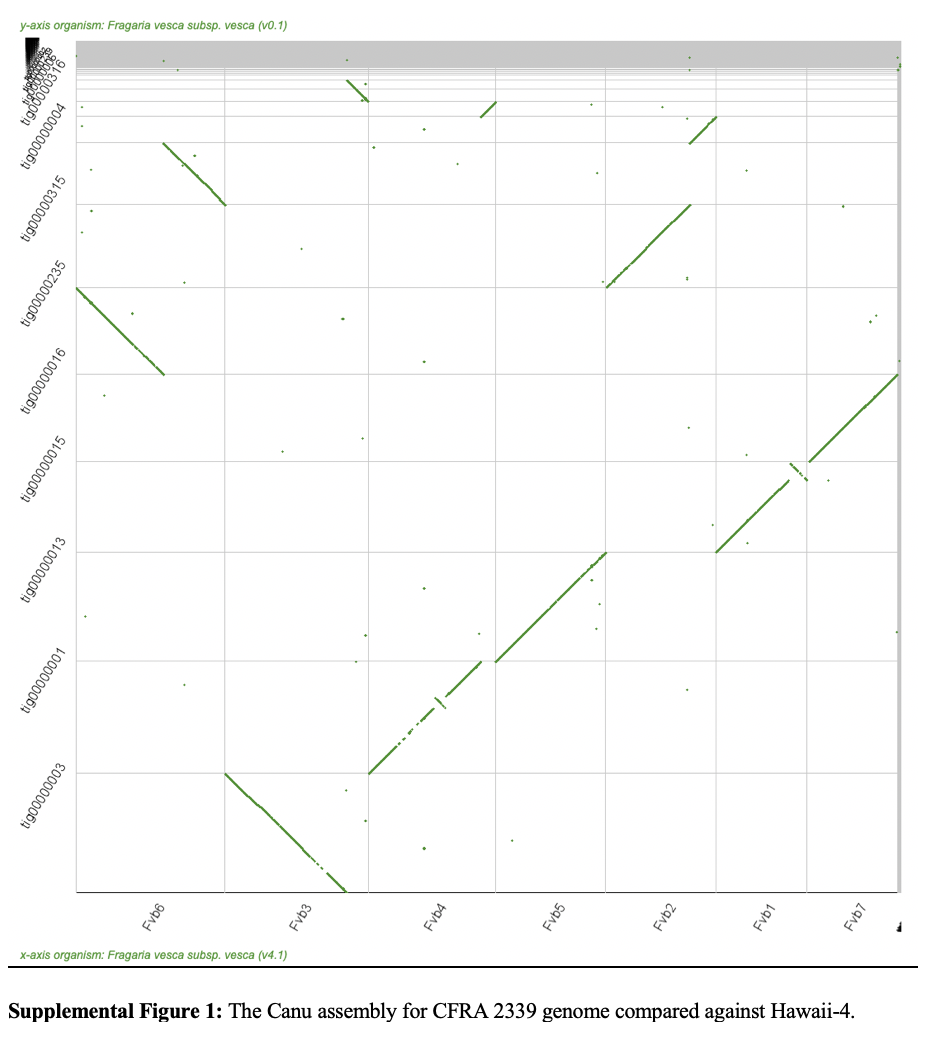

Supplement: Supplementary file 6 [file Image_1.png]

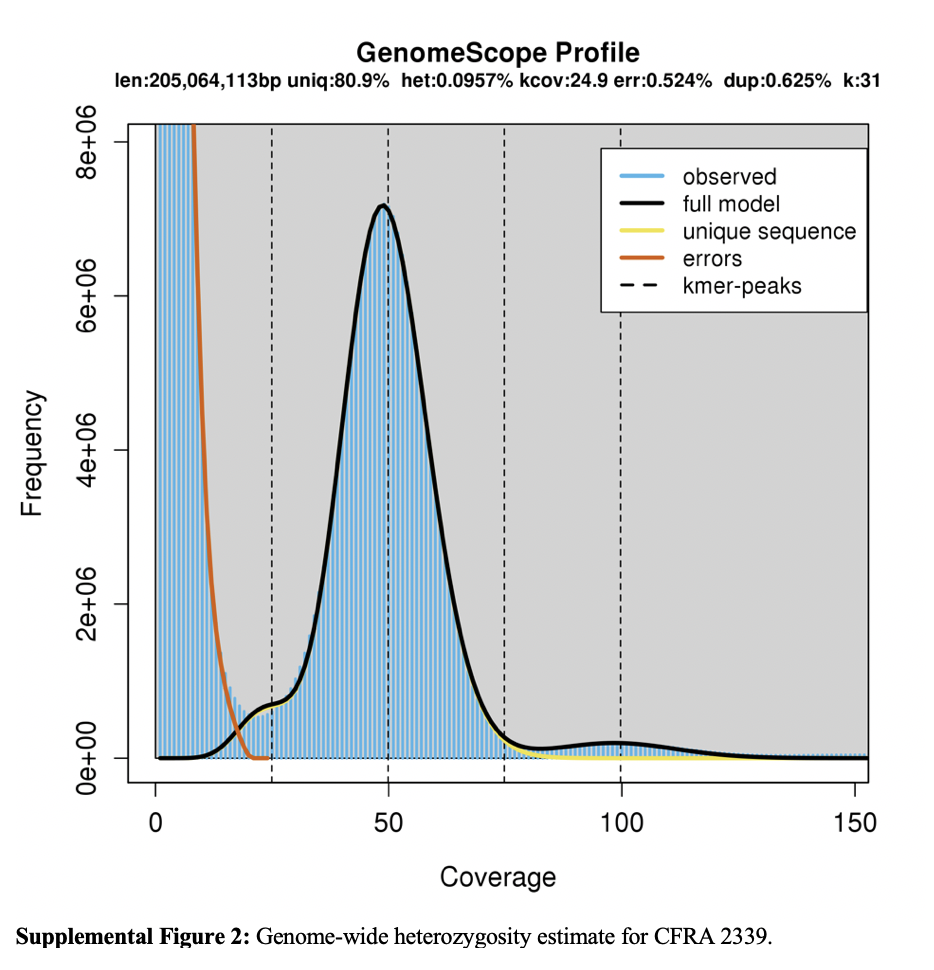

Supplement: Supplementary file 7 [file Image_2.png]

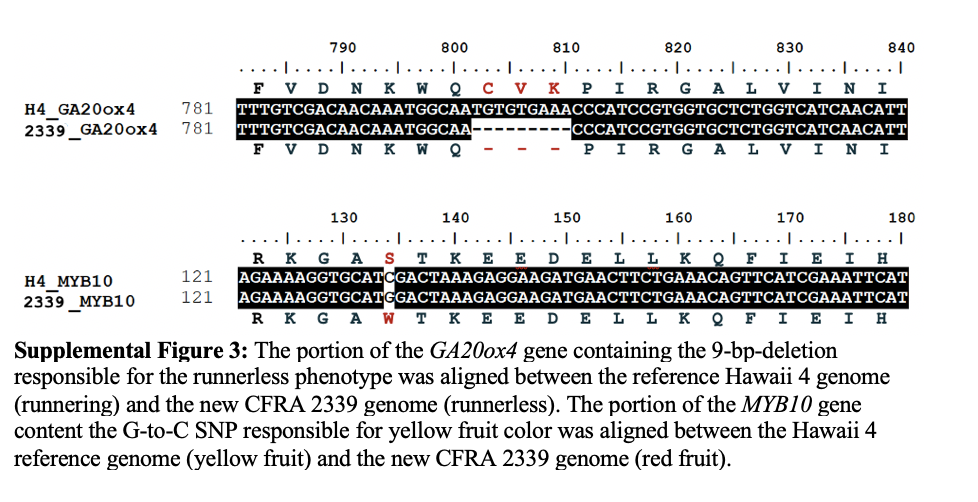

Supplement: Supplementary file 8 [file Image_3.png]
