# Supplementary material for: Chromosome-Scale Genome for a Red-Fruited, Perpetual Flowering and Runnerless Woodland Strawberry (Fragaria vesca)
Source: Front Genet. 2021 Jul 16;12:671371. doi: 10.3389/fgene.2021.671371 (PMC8323839; doi:10.3389/fgene.2021.671371)
Supplement: Supplementary file 4 [file Table_4.docx]

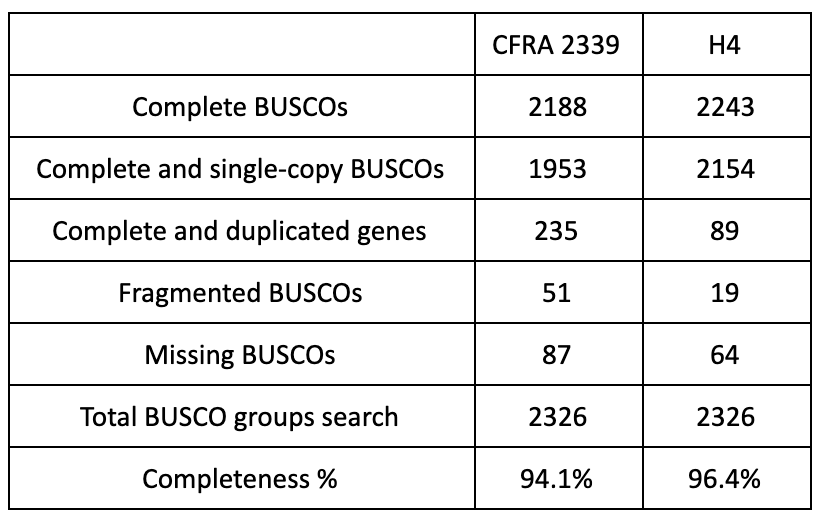


**Supplemental Table 4:** Summarized BUSCO score using the eudicot database (eudicots_odb10) of CFRA 2339 and Hawaii-4.
