# Supplementary material for: Chromosome-Scale Genome for a Red-Fruited, Perpetual Flowering and Runnerless Woodland Strawberry (Fragaria vesca)
Source: Front Genet. 2021 Jul 16;12:671371. doi: 10.3389/fgene.2021.671371 (PMC8323839; doi:10.3389/fgene.2021.671371)
Supplement: Supplementary file 5 [file Table_5.docx]

| TE Super-family | | Count | Coverage (Mb) | Fraction of genome (%) |
| --- | --- | --- | --- | --- |
| Class I | LTR/*Copia* | 12538 | 9.30 | 4.05% |
|  | LTR/*Gypsy* | 13729 | 13.23 | 5.76% |
|  | LTR/Unknown | 22633 | 14.01 | 6.10% |
|  | **Total Class I** | **48900** | **36.53** | **15.91%** |
| Class II | CACTA | 14859 | 8.02 | 3.50% |
|  | Mutator | 25576 | 7.79 | 3.40% |
|  | *PIF/Harbinger* | 6825 | 2.33 | 1.01% |
|  | *Tc1/mariner* | 820 | 0.23 | 0.10% |
|  | *hAT* | 11007 | 4.02 | 1.75% |
|  | *Helitron* | 24959 | 9.14 | 3.98% |
|  | **Total Class II** | **84046** | **31.54** | **13.74%** |
| **Total TEs** | | **132946** | **68.07** | **29.66%** |

**Supplemental Table 5**: Summarized transposable elements (TE) composition for CFRA 2339.
